# Editorial: The outcomes of pollutants on glia

**DOI:** 10.3389/fncel.2024.1444344

**Published:** 2024-07-10

**Authors:** Gabriela P. Arrifano, Marcus Augusto-Oliveira, Marie-Eve Tremblay, Maria Elena Crespo-Lopez

**Affiliations:** ^1^Laboratório de Farmacologia Molecular, Instituto de Ciências Biológicas, Universidade Federal do Pará, Belém, Brazil; ^2^Division of Medical Sciences, University of Victoria, Victoria, BC, Canada

**Keywords:** pollution, glia, intoxication, heavy metals, pesticides, xenobiotics, neurotoxicity

The global environment is experiencing profound challenges, with pollution being the largest environmental cause of disability and death, resulting in ~9 million deaths per year (Fuller et al., 2022). Pollutants reach humans and animals through several routes including air, freshwater, and ocean contamination and food chain. The impacts of pollutants are ubiquitous, directly and indirectly affecting people living both in urban centers and remote locations, including those of riverine settings, and indigenous people (Fernández-Llamazares et al., 2020; Crespo-Lopez et al., 2021).

Among the health problems caused by pollutants, neurological outcomes are of special concern due to several reasons including (i) difficulty of early detection; (ii) management complexity, generally with pharmacological approaches of narrow therapeutic index; (iii) possibility of permanent damage depending on the brain area affected; (iv) risk of impaired neurodevelopment in children, leading to long-lasting disabilities, and consequently also having social and economic impacts (Augusto-Oliveira et al., 2021a; Theron et al., 2022).

Because most pollutants can cross the blood-brain barrier, they enter the brain and cause cellular and molecular disruption. By affecting redox systems, neurotransmitter and glucose metabolism, calcium signaling, and cytokine production/release, among other mechanisms, they eventually lead to motor, visual, cognitive and mood disorders, as well as contribute to neurodegenerative diseases. Among brain cells, glia account for roughly 50%, depending on the context, being involved in all brain functions from neurogenesis and synaptogenesis to homeostasis and defense (Augusto-Oliveira et al., 2020, 2021b). These cells orchestrate neural plasticity in response to pollutant neurotoxicity, driving beneficial or deleterious responses depending on numerous factors including duration and frequency of pollutant exposure, body burden, and age, among others (Arrifano et al., 2021; de Paula Arrifano et al., 2023; Leal-Nazaré et al., 2024).

The four articles included in this Research Topic reflect the broad scope of the current research investigating glial involvement in the neurological outcomes induced by pollutants.

Regarding the involvement of glia in neurodegenerative diseases such as multiple sclerosis, Alzheimer's and Parkinson's diseases, and amyotrophic lateral sclerosis associated with metal toxicity, Pamphlett and Bishop raised an interesting and elegant hypothesis: toxic metals damage the *locus coeruleus*, disrupting the blood-brain barrier by reducing noradrenaline signaling. This would allow circulating pollutants to enter astrocytes, impairing astroglial functions, and being transferred to neurons and oligodendrocytes, thereby leading to multifocal cellular dysfunction. According to this hypothesis, the resultant neurological disorder would depend on numerous aspects including (i) identity of neurons affected in the *locus coeruleus*; (ii) genetic susceptibility related to metal uptake, cytotoxicity and clearance; (iii) individual age, duration and frequency of metal exposure, and (iv) co-exposure to several pollutants simultaneously. The hypothesis is supported by evidence from human and animal studies, especially pertaining to mercury and silver intoxication, and the link between metal toxicity and clinicopathological features shared between multiple sclerosis, Alzheimer's and Parkinson's diseases, and amyotrophic lateral sclerosis.

Besides metals, fluoride, a non-metal widely utilized in drinking water and various dental products to prevent dental caries and tooth decay, received considerable attention as a neurotoxicant, although the underlying mechanism of action remains controversial. Puty et al. investigated the molecular features of fluoride neurotoxicity in the U87 glia-like cell line. Using transcriptomic and metabolomic approaches, the authors evaluated the effects of exposure to two different fluoride concentrations (0.095 and 0.22 μg/ml) for 10 days. This revealed that fluoride interferes with genic expression related to cellular metabolism, protein modification and pathways regulating cell death such as mitogen-activated protein kinase cascade. The findings were confirmed by proteomic analysis, showing changes in energy metabolism and cytoskeletal elements, shedding light onto the possible mechanisms underlying fluoride toxicity in glia-like cells.

Investigating the behavior of glial cells is paramount to understand how these cells can influence brain protection or damage resulting from pollutant intoxication. Kim et al. generated a new conditional transgenic mouse model that allows to induce apoptosis via Cre-dependent active caspase-3 during normal physiological conditions. By transiently triggering apoptotic cell death of hippocampal astrocytes, the authors have induced astrocyte reactivity (morphological, molecular, and functional changes in response to pathological context) and astrocytogenesis in adult mice. Although transient astrocyte depletion caused cognitive dysfunction during the 1st week, the animals recovered during weeks 2 and 4, showing astrocyte number and function restoration, with no significant sign of neurotoxicity. Thus, the developed mouse model emerges as a valuable strategy to study gliogenesis across different brain areas, with the potential to understand astroglial involvement in pollutant-induced neurotoxicity.

Among the non-pharmacological alternatives to prevent or mitigate pollutant-induced neurotoxicty, the diet emerges as a powerful tool, with significant impacts on brain function. Analyzing how the diet affects glial function is critical to understand its involvement in a neurotoxic context. Butler et al. have investigated how dietary fatty acids impacts cellular functions in the brain. Using microglia-like (BV2) and neuron-like (HippoE-14) cell lines, the authors have found that palmitate, an abundant saturated fat component of high-fat diets, differently impacts phagocytosis, inflammatory gene expression and mitochondrial activity. In fact, synaptoneurosomes isolated from high-fat diet-fed mice were engulfed by BV2 microglia-like cells at a faster rate than synaptoneurosomes isolated from chow-fed mice. This is an interesting data suggesting that high-fat diet alters synaptic signaling to increase microglial phagocytic activity. Also, an increased complement protein expression on synaptoneurosomes isolated from the hippocampus of high fat-fed mice was detected. Palmitate reduced mitochondrial activity both in microglia-like and neuron-like cells, which was not mitigated with omega-3 fatty acid (decosahexanoic acid) pretreatment, suggesting this omega-3 fatty acid exerts its protective actions downstream of mitochondrial functions.

Collectively, these studies demonstrate the involvement of glial cells in mediating both pollutants-induced neurotoxicity and outcomes of the diet, a modifiable factor critical to prevent or mitigate such neurotoxicity. Considering that pollution is a global threat, with deleterious consequences not only for health but also for socio-economic aspects, it is imperative to continue efforts to understand how to prevent and mitigate the neurological consequences of pollution exposure. This volume contributes to improve our knowledge of the glial role in pollutants toxicity.

## Author contributions

GA: Writing – original draft, Writing – review & editing. MA-O: Writing – original draft, Writing – review & editing. M-ET: Writing – original draft, Writing – review & editing. MC-L: Writing – original draft, Writing – review & editing.
